# Focus on Diagnosis and Prognosis to Guide Timing of Intervention in Valvular Heart Disease

**DOI:** 10.1007/s11886-022-01754-w

**Published:** 2022-08-04

**Authors:** Jan Stassen, Xavier Galloo, Pieter van der Bijl, Jeroen J. Bax

**Affiliations:** 1 Department of Cardiology, Heart Lung Center, Leiden University Medical Center, Albinusdreef 2, 2300 RC Leiden, The Netherlands; 2 Department of Cardiology, Jessa Hospital, Stadsomvaart 11, 3500 Hasselt, Belgium; 3 Department of Cardiology, Universitair Ziekenhuis Brussel (UZ Brussel), Vrije Universiteit Brussel (VUB), Laarbeeklaan 101, 1090 Brussels, Belgium; 4 Turku Heart Center, University of Turku and Turku University Hospital, Kiinamyllynkatu 4-8, 20520 Turku, Finland

**Keywords:** Valvular heart disease, Aortic stenosis, mitral regurgitation, Tricuspid regurgitation, Multimodality imaging

## Abstract

**Purpose of Review:**

The present article reviews the role of multimodality imaging to improve risk stratification and timing of intervention in patients with valvular heart disease (VHD), and summarizes the latest developments in transcatheter valve interventions.

**Recent Findings:**

Growing evidence suggests that intervention at an earlier stage may improve outcomes of patients with significant VHD. Multimodality imaging, including strain imaging and tissue characterization with cardiac magnetic resonance imaging, has the ability to identify early markers of myocardial damage and can help to optimize the timing of intervention. Transcatheter interventions play an increasing role in the treatment of patients who remain at high surgical risk or present at a late stage of their disease.

**Summary:**

Multimodality imaging identifies markers of cardiac damage at an early stage in the development of VHD. Together with technological innovations in the field of percutaneous valvular devices, these developments have the potential to improve current management and outcomes of patients with significant VHD.

## Introduction

Valvular heart disease (VHD) is a major health problem, affecting more than 2% of the general population. It is associated with a decreased quality of life and an increased risk of cardiovascular events, including heart failure hospitalizations and mortality [1]. Because of the prolonged survival of patients with heart failure and the aging population, many patients with significant VHD are considered inoperable or are at high surgical risk. Less invasive, transcatheter valvular interventions have therefore received increased attention. Transcatheter aortic valve replacement (TAVR) has proven its value in high-risk patients and has evolved from a challenging intervention to a standardized and streamlined procedure with a clear trend towards the expansion of indications to intermediate and low-risk groups. Similarly, major trials utilizing transcatheter therapies for secondary mitral regurgitation (MR) have recently been completed and transcatheter edge-to-edge repair now offers new treatment options for patients with secondary MR who are inoperable or at high surgical risk. The successes of transcatheter therapies for left-sided VHD have fueled the development of novel transcatheter devices for the treatment of right-sided VHD, and studies on transcatheter edge-to-edge repair in patients with secondary tricuspid regurgitation (TR) are ongoing. These technological innovations, together with a better understanding of the mechanisms underlying VHD, have led to major improvements in the management of patients with significant VHD.

Timely diagnosis and adequate risk stratification remain essential in the decision-making process of patients with significant VHD. According to the European and American guidelines, left ventricular (LV) ejection fraction (EF) and the onset of symptoms remain the most important parameters when deciding to refer a patient with left-sided VHD for intervention [2, 3]. Similarly, right ventricular (RV) size/function and symptoms also play a pivotal role when referring a patient with right-sided VHD for intervention [2, 3]. However, deciding whether the onset of symptoms is caused by the underlying VHD is challenging, especially because most patients also have concomitant (non-)cardiovascular comorbidities (i.e., coronary artery disease, pulmonary disease, limited mobility). In addition, LVEF and RVEF often remain preserved for a long time despite ongoing, progressive cardiac remodeling. For example, in aortic stenosis (AS), pressure overload induces a hypertrophic remodeling response to reduce wall stress, which maintains LVEF. This response may eventually lead to ischemia and LV myocardial fibrosis, even though LVEF is still preserved. In patients with MR or TR, EF calculates the total volume of blood that is displaced from the ventricle, not taking into consideration the percentage of blood that is directed backwards to the atrium (and therefore does not contribute to cardiac output). LVEF and RVEF therefore often appear falsely preserved (or even supranormal) while eccentric remodeling due to volume overload is already occurring. These observations underscore the need to identify earlier risk markers of myocardial damage beyond symptoms and EF, to improve risk stratification and optimize the timing of intervention in patients with VHD. Evidence supporting the prognostic value of speckle-tracking echocardiography–derived strain imaging and cardiac magnetic resonance (CMR)–based tissue characterization is increasing and these novel imaging markers are finding their way into clinical practice.

In this review, we discuss how multimodality imaging can help to improve the management of VHD and highlight the latest developments of transcatheter interventions in AS, as well as in secondary MR and TR.

## Latest Developments in the Management of Aortic Stenosis

AS is the most common VHD worldwide, and its prevalence is rising rapidly due to the aging population [1, 4]. Current guidelines recommend AVR when patients with severe AS become symptomatic or when LV systolic function is impaired (i.e., LVEF < 50%) [2, 3]. In contrast, an expectant but vigilant approach has been proposed in asymptomatic patients who show no adverse prognostic features [2, 3]. This strategy, however, has been questioned in recent years with the observation that the prognosis of conservatively managed, asymptomatic patients with severe AS may not be benign [5]. In addition, a study comparing a conservative treatment strategy with early surgical AVR demonstrated that early surgical AVR was associated with improved survival (i.e., lower cardiac mortality and sudden cardiac death) in asymptomatic patients with very severe AS [6]. Similarly, the recently performed Aortic Valve replAcemenT versus conservative treatment in Asymptomatic seveRe aortic stenosis (AVATAR) trial showed that asymptomatic patients with severe AS who underwent early surgical AVR had a significant reduction in the primary endpoint (a composite of all-cause death, acute myocardial infarction, stroke or unplanned heart failure hospitalization) compared to patients who underwent conservative treatment [7]. These studies raise the question whether waiting for symptoms to occur or LVEF to decline may worsen prognosis in asymptomatic patients who are currently being treated conservatively.

To optimize risk stratification and management of asymptomatic patients with severe AS, a more thorough understanding of the underlying pathophysiology is imperative. Severe AS causes pressure overload of the LV, whereafter LV hypertrophy develops to reduce wall stress and maintain cardiac output. With the development of LV hypertrophy, myocardial oxygen demand increases while coronary flow reserve decreases due to concomitant microvascular dysfunction, low coronary perfusion pressure, and reduced diastolic perfusion time [8]. This oxygen demand/supply mismatch triggers subendocardial ischemia [9], which leads to myocyte degeneration with irreversible cell loss and the formation of myocardial fibrosis [10]. As such, significant cardiac remodeling may already occur in patients with severe AS in whom LVEF is still preserved. From this perspective, the identification of early markers of myocardial damage (i.e., before a reduction in LVEF is observed) that can improve risk stratification and allow early referral for intervention is a priority. Besides important risk factors, including hemodynamic response to exercise, AS severity by jet velocity, rate of AS progression, pulmonary hypertension, and elevated brain natriuretic peptides, increasing attention has been given to speckle-tracking echocardiography–derived global longitudinal strain (GLS) measurements and the detection of myocardial fibrosis with CMR.

**Fig. 1:**
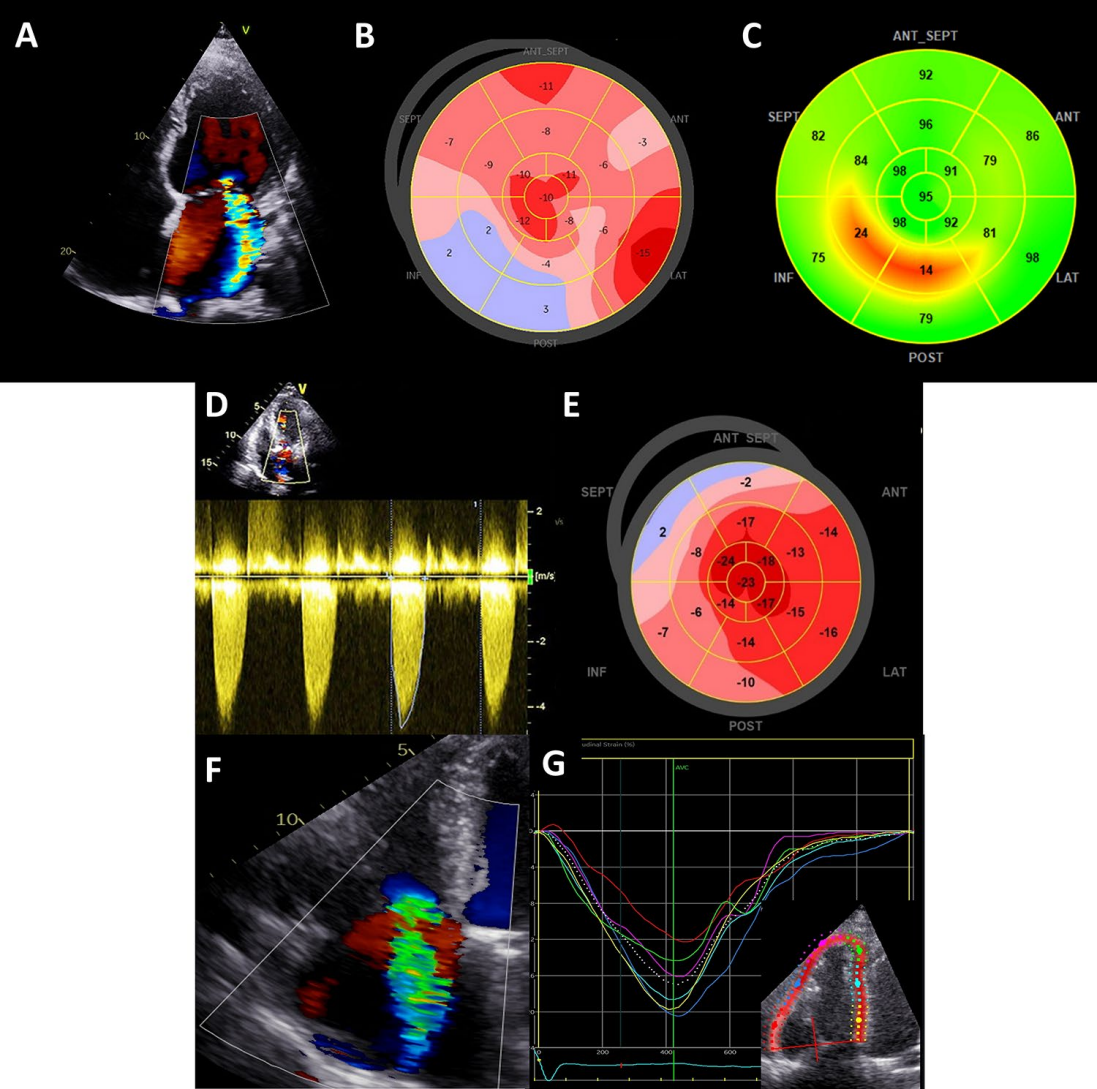
Speckle-tracking strain imaging in patients with valvular heart disease. The figure shows three examples of patients with valvular heart disease and the use of 2D speckle-tracking strain echocardiography. Patient 1 (panels **A**, **B**, **C**): a 67-year-old male patient with ischemic cardiomyopathy and non-viable myocardium after right coronary artery (RCA) myocardial infarction, presented with severe secondary mitral regurgitation due to tethering of the posteromedial papillary muscle (panel **A**). Left ventricular global longitudinal strain (GLS) was − 7.5%, with dyskinesia (blue color) in the RCA territory on a parametric GLS map (panel **B**). Impaired myocardial work efficiency can be seen in the RCA territory on a parametric map (panel **C**). Patient 2 (panels **D**, **E**): a 75-year-old male patient presented with severe aortic stenosis (aortic valve area 0.75 cm.^2^, peak aortic jet velocity 4.25 m/s, mean gradient 51 mmHg) (panel **D**). Although the left ventricular ejection fraction was preserved (56%), the left ventricular GLS was reduced (− 13.5%) (panel **E**). Patient 3 (panels **F**, **G**): a 69-year-old female patient presented with severe, secondary TR. Although tricuspid annular plane systolic excursion was preserved (21 mm) (panel **F**), right ventricular free wall strain was reduced (− 16%) (panel **G**)

Reduced LV GLS is a more sensitive marker of impaired LV contractile function than LVEF and shows a strong association with the presence of myocardial fibrosis on CMR [11, 12] (Fig. 1). Evidence is accruing that LV GLS may have important prognostic implications in asymptomatic patients with severe AS and preserved LVEF. In a recent meta-analysis, including 1067 patients with asymptomatic severe AS and preserved LVEF, a LV GLS value < 14.7% (absolute value) was associated with a 2.5-fold higher risk of all-cause mortality [13].

**Fig. 2:**
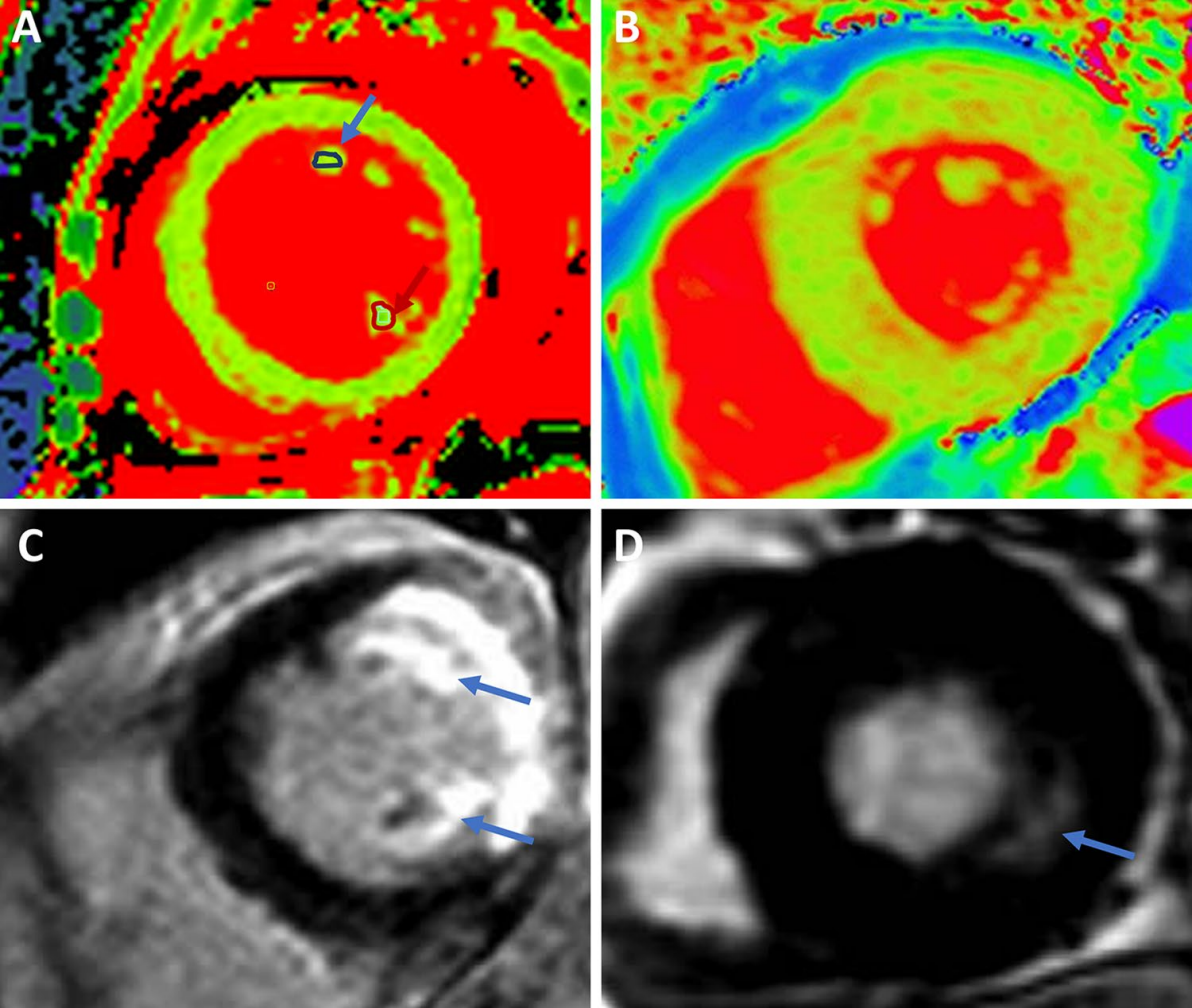
Tissue characterization with cardiac magnetic resonance imaging. The figure shows four examples of patients with valvular heart disease and the use of cardiac magnetic resonance imaging to assess left ventricular myocardial fibrosis. Patient 1: a 44-year-old female with prior myocarditis and a left ventricular ejection fraction of 21% presented with severe secondary mitral regurgitation. Regions of interest for native T1 time have been drawn inside both papillary muscles (blue and red arrow) and show an elevated native T1 time of the anterolateral (1184 ms) and the posteromedial papillary muscles (1148 ms) (panel **A**). Patient 2: a 78-year-old male patient presented with severe aortic stenosis and preserved left ventricular ejection fraction of 58%. The T1 time of the left ventricular myocardium is increased (984 ms) (panel **B**). Patient 3: a 54-year-old male patient presented with a lateral myocardial infarction and necrosis of both papillary muscles (*blue arrows*), causing severe mitral regurgitation (panel **C**). Patient 4: a 76-year-old female patient presented with severe aortic stenosis. Cardiac magnetic resonance imaging shows the presence of mid-wall replacement fibrosis (*blue arrow*) (panel **D**)

CMR can provide myocardial tissue characterization, enabling the non-invasive assessment of LV fibrosis (Fig. 2). LV fibrosis can be divided into 2 types: reactive interstitial fibrosis and focal replacement fibrosis. Replacement fibrosis in AS reflects a more advanced disease and histopathological studies have demonstrated that it is one of the main factors driving the transition from LV hypertrophy to heart failure [10]. Replacement fibrosis in AS typically presents in a non-infarct, “mid-wall” pattern of late gadolinium enhancement (LGE) (Fig. 2). This typical pattern can help to differentiate scarring due to AS from other causes (e.g., cardiac amyloidosis, myocardial infarction). In 674 patients with severe AS undergoing AVR, the presence of LGE was an independent and powerful predictor of worse outcomes, correlating with mortality in a dose-dependent manner (every 1% increase in LGE increased mortality hazard by 11% and cardiovascular mortality hazard by 8%) [14]. Reactive interstitial fibrosis occurs in the earlier stages of AS (before replacement fibrosis) and reflects excess collagen deposition in the extracellular matrix. Two parameters (T1 mapping and extracellular volume quantification) have been shown to accurately quantify the amount of interstitial fibrosis on CMR (Fig. 2). In 440 patients with severe AS undergoing AVR, Everett and colleagues [15] demonstrated that extracellular volume fraction provided the strongest prognostic information and was superior to LVEF, with every 1% increase being independently associated with a 10% increase in the risk of all-cause mortality. The Evaluation of Transcatheter Aortic Valve Replacement Compared to Surveillance for Patients With Asymptomatic Severe Aortic Stenosis (EARLY TAVR) trial (NCT03042104) and the Early Valve Replacement Guided by Biomarkers of LV Decompensation in Asymptomatic Patients With Severe AS (EVOLVED) trial (NCT03094143) are currently investigating whether early AVR benefits asymptomatic patients with severe AS. The results of these studies are eagerly awaited, and have the potential to change clinical practice.

Recently, several observational studies have shown that moderate AS is also associated with a considerable risk of adverse cardiovascular events, including death [16, 17•]. These studies support the concept that anatomic indices of aortic valve area and pressure gradients are probably insufficient to describe AS severity and impact on LV performance. AS stenosis may better be approached as a continuous variable, with each incremental increase imposing an increased pressure load on the LV. The identification of early markers of myocardial damage may therefore also improve the risk stratification of patients with moderate AS. Hayward and colleagues [18] showed that the degree of LV systolic dysfunction assessed by LV GLS, rather than LVEF, was the major determinant of outcome in 169 patients with moderate AS and reduced LVEF. Stassen et al. demonstrated that LV GLS was independently associated with all-cause mortality in patients with moderate AS and preserved LV. In addition, patients with moderate AS and preserved LVEF but impaired LV GLS (<16%, absolute value) had equally worse outcomes compared to those with reduced LVEF [19]. In 143 patients with moderate (40%) or severe (60%) AS, 50% of the patients with mid-wall LGE had moderate AS [20]. In addition, more than half of the patients with mid-wall fibrosis who died in the same study had moderate AS [20]. This raises the question whether patients with moderate AS showing signs of cardiac damage are being referred too late and would benefit from early AVR. The Transcatheter Aortic Valve Replacement to Unload the Left Ventricle in Patients With Advanced Heart Failure (TAVR UNLOAD) (NCT02661451) and the Prospective, Randomized, Controlled Trial to Assess the Management of Moderate Aortic Stenosis by Clinical Surveillance or Transcatheter Aortic Valve Replacement (PROGRESS) trial (NCT04889872) will investigate whether transcatheter AVR could improve outcomes in these patients.

## Latest Developments in the Management of Secondary Mitral Regurgitation

Secondary MR occurs as a result of LV adverse remodeling and papillary muscle tethering and/or dyssynchrony in patients with LV disease. The presence of secondary MR has been linked to all-cause mortality and heart failure hospitalizations [21–23]. However, most data regarding the impact of secondary MR on prognosis originate from retrospective studies. Since multivariable models may fail to take into account all confounders, the true independent contribution of secondary MR to outcomes remains unresolved. All patients with secondary MR and heart failure with a reduced LVEF should first of all receive optimal, guideline-directed medical therapy, including cardiac resynchronization therapy. The benefit of performing an additional intervention to address secondary MR is the subject of ongoing debate. Traditionally, restrictive annuloplasty has been performed surgically in selected patients, but suffers from the invasive nature of the procedure. In addition, a significant number of patients experience residual or recurrent secondary MR after surgery. No clear prognostic benefit has ever been demonstrated for surgical mitral valve repair in secondary MR, although symptomatic benefit, reverse LV remodeling, and improved LV systolic function have been documented [24, 25]. A trend towards improved survival has been shown when a mitral repair was performed in the context of coronary artery bypass surgery in patients with a LVEF ≤ 35% [26]. Results from the Cardiovascular Outcomes Assessment of the MitraClip Percutaneous Therapy for Heart Failure Patients with Functional Mitral Regurgitation (COAPT) trial suggest that all-cause mortality and heart failure hospitalization can be reduced by selective application of percutaneous, edge-to-edge mitral valve repair [27••]. However, approximately 50% of patients allocated to the treatment arm of the COAPT trial were still admitted for heart failure hospitalization or died within 2 years after the intervention [28]. These patients therefore remain at high risk, despite the performance of percutaneous mitral valve repair. Because secondary MR is independently associated with worse outcomes and evidence exists for symptomatic and prognostic benefit after percutaneous repair, the question remains if improved risk stratification and earlier intervention can improve the results of interventional therapies, especially transcatheter repair. A number of emerging imaging biomarkers represent independent risk factors in patients with secondary MR and have the potential to refine the selection of patients, as well as the timing of transcatheter mitral valve transcatheter repair.

The LV regurgitant volume/LV end-diastolic volume ratio represents the absolute severity of regurgitation to the degree of LV remodeling, and was independently associated with all-cause mortality in a study of 379 patients with significant secondary MR [29]. Expressing secondary MR as the ratio of the effective regurgitant orifice area/LV end-diastolic volume is an alternative approach to express the proportionality of regurgitation in relation to the degree of LV remodeling. Disproportionate secondary MR (i.e., more severe MR and less severe LV remodeling) may respond better to transcatheter therapies than pharmacologic therapy alone [30]. The outcome of secondary MR may be modulated by underlying myocardial scar tissue. In a study of 441 patients who underwent LGE-CMR, secondary MR with a regurgitant fraction > 30% was associated with outcomes only in the presence of myocardial scar [31]. The link between scar tissue and outcomes may be explained by the presence of LV viability, which predicted improvement of secondary MR in response to coronary artery bypass surgery [32] (Fig. 2). Not only replacement fibrosis (detected by LGE-CMR) but also interstitial LV fibrosis may impact on secondary MR. Papillary muscle native T1 time has been correlated with secondary MR regurgitant fraction in a population of patients with non-ischemic dilated cardiomyopathy (Fig. 2) [33]. In addition to LV structure, LV functional parameters may have prognostic value in risk-stratifying patients with secondary MR. In a study of 650 patients with secondary MR, LV GLS was found to be an independent predictor of all-cause mortality, while LVEF was not (Fig. 1) [34]. Non-invasive LV myocardial work parameters have also been linked to long-term survival in patients with significant secondary MR (Fig. 1) [35]. In a large cohort of patients who were investigated for suspected coronary artery disease, LV subpapillary ischemia on pharmacologic stress CMR was associated with the severity of secondary MR, independent of scar tissue [36]. Not only LV but also left atrial function may be important in characterizing the effect of secondary MR. Left atrial reservoir strain was independently associated with all-cause mortality in a study of 666 patients with significant secondary MR [37].

The interventional treatment of secondary MR is undergoing rapid evolution. Percutaneous, edge-to-edge mitral valve repair has demonstrated symptomatic and prognostic benefits in selected patients, but may not be well suited to all mitral valve anatomies. Edge-to-edge repair does not directly address mitral annular dilation, for which surgical mitral annuloplasty has been a mainstay [38]. A multitude of percutaneous mitral valve replacement devices are in development that have the potential to correct the valvular as well as the annular components of secondary MR [38]. Percutaneous mitral valve replacement devices may also prove useful in the management of failed percutaneous edge-to-edge repair, or when suboptimal results have been obtained [38].

## Latest Developments in the Management of Secondary Tricuspid Regurgitation

Significant (moderate or severe) TR is frequently observed in heart failure patients, with a prevalence of up to 23% [39]. Despite this high prevalence, TR has long been considered an innocent bystander when it comes to risk stratification and treatment of these patients. More recent data, however, have shown an independent association between TR severity and outcomes, even after adjusting for left-sided VHD, LVEF, pulmonary hypertension, and right heart failure [40•]. The question therefore remains if tricuspid valve (TV) intervention can improve outcomes in well-selected patients with significant TR. Previously, the idea was that TR severity reliably improves once the left-sided pathology (i.e., LV systolic dysfunction or significant VHD) was addressed and little attention was given to TV surgery for secondary TR [41]. However, more recent data showed that unoperated TR at the time of left-sided valve surgery will progress to significant TR in up to 33% of patients and is associated with worse outcomes [42]. In addition, these data also showed that concomitant TV surgery did not increase perioperative mortality and was associated with right-sided reverse remodeling and improved long-term outcomes [42]. These observations have led to an increased interest in TV surgery. However, optimal timing for surgical intervention in patients with severe TR remains challenging and the advantage of TV surgery over medical therapy remains a matter of debate. In a recent randomized controlled trial including patients undergoing mitral valve surgery, those who also received TV annuloplasty had a lower incidence of primary endpoint events compared to those who underwent mitral valve surgery alone at 2-year follow-up [43]. However, this reduction was mainly driven by less frequent progression to severe TR (not by mortality). Whether this reduced progression of TR results in long-term clinical benefit needs to be proven with longer-term follow-up data [43]. In a retrospective analysis including patients with isolated severe TR, TV surgery was not associated with improved long-term survival when compared to medical management alone after accounting for immortal time bias [44]. Furthermore, isolated TV surgery for severe TR has been associated with a high morbidity and in-hospital mortality, with the latest data from a French registry showing an in-hospital mortality of 10% [45]. However, this high in-hospital mortality rate was mainly attributed to multiple comorbidities and late referral for surgery. Indeed, current European and American guidelines only recommend TV surgery in patients with severe secondary TR who are symptomatic or show RV dilation (unless patients also undergo left-sided surgery) [2, 3]. However, significant TR may remain asymptomatic for an extended period of time, even though it has already led to progressive RV dilation and dysfunction, which will eventually cause symptoms of right heart failure and increase mortality [40•]. The question therefore remains if tricuspid valve (TV) intervention can improve outcomes in well-selected patients with significant TR. Previously, the idea was that TR severity reliably improves once the left-sided pathology (i.e., LV systolic dysfunction or significant VHD) was addressed and little attention was given to TV surgery for secondary TR [41]. However, more recent data showed that unoperated TR at the time of left-sided valve surgery will progress to significant TR in up to 33% of patients and is associated with worse outcomes [42]. In addition, these data also showed that concomitant TV surgery did not increase perioperative mortality and was associated with right-sided reverse remodeling and improved long-term outcomes [42]. These observations have led to an increased interest in TV surgery. However, optimal timing for surgical intervention in patients with severe TR remains challenging and the advantage of TV surgery over medical therapy remains a matter of debate. In a recent randomized controlled trial including patients undergoing mitral valve surgery, those who also received TV annuloplasty had a lower incidence of primary endpoint events compared to those who underwent mitral valve surgery alone at 2-year follow-up [43]. However, this reduction was mainly driven by less frequent progression to severe TR (not by mortality). Whether this reduced progression of TR results in long-term clinical benefit needs to be proven with longer-term follow-up data [43]. In a retrospective analysis including patients with isolated severe TR, TV surgery was not associated with improved long-term survival when compared to medical management alone after accounting for immortal time bias [44]. Furthermore, isolated TV surgery for severe TR has been associated with a high morbidity and in-hospital mortality, with the latest data from a French registry showing an in-hospital mortality of 10% [45]. However, this high in-hospital mortality rate was mainly attributed to multiple comorbidities and late referral for surgery. Indeed, current European and American guidelines only recommend TV surgery in patients with severe secondary TR who are symptomatic or show RV dilation (unless patients also undergo left-sided surgery) [2, 3]. However, significant TR may remain asymptomatic for an extended period of time, even though it has already led to progressive RV dilation and dysfunction, which will eventually cause symptoms of right heart failure and increase mortality [40•, 46]. Galloo et al. [47] demonstrated that the majority of patients with significant TR are indeed referred at a late stage, having symptoms and signs of right heart failure, which was associated with worse 5-year mortality rates. Moreover, when symptoms are present, diuretic treatment effectively improves these symptoms, leading to a low referral rate for intervention [48]. Currently, only 5% of patients with significant TR are referred for surgery [48].

**Fig. 3:**
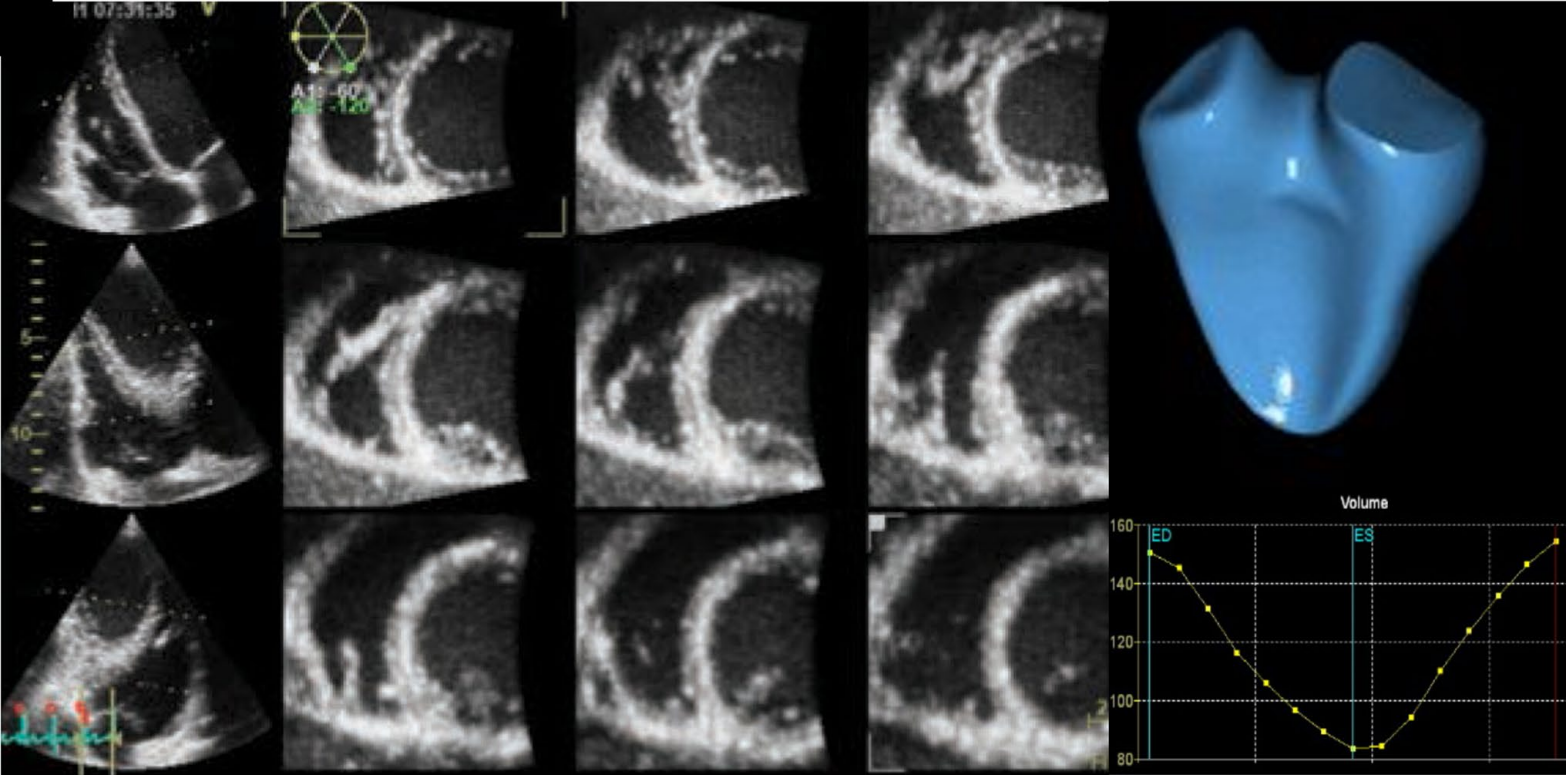
Three-dimensional echocardiographic measurements of the right ventricular volumes and ejection fraction. A 72-year-old man presented with severe secondary TR. Two-dimensional echocardiography showed a non-dilated right ventricle (basal diameter 39 mm) and a preserved tricuspid annular plane systolic excursion (18 mm). In contrast, three-dimensional echocardiography shows a mildly dilated right ventricle (end-diastolic volume 91 ml/m.^2^) with an impaired right ventricular ejection fraction (42%)

Although the optimal timing for TV surgery remains a conundrum, patients should probably be referred for TV surgery at an earlier stage, prior to the onset of symptoms, severe RV systolic dysfunction, and end-organ damage. Evaluation of the right heart plays a central role in optimizing the risk stratification of patients undergoing TV surgery. Dietz et al. [49] showed that patients with significant secondary TR had worse outcomes in the presence of RV systolic dysfunction, regardless of the presence of RV dilation. In contrast, patients showing RV dilation, but preserved RV systolic function, had comparable outcomes to those showing no RV remodeling [49]. This observation underscores the importance of early referral, before RV systolic dysfunction occurs. Two-dimensional echocardiography is considered the first-line imaging technique for the evaluation of RV remodeling. However, it is limited by its inability to accurately assess the complex three-dimensional geometry of the RV. Muraru et al. [50] showed the incremental value of three-dimensional echocardiography to evaluate the relationship between the TV annulus area and right atrial/RV volumes in patients with TR (Fig. 3). Moreover, the coaptation zone in secondary TR is often non-circular and evaluation of the vena contracta width with two-dimensional imaging is often inaccurate. In this setting, quantification of the vena contracta using three-dimensional planimetry may be superior [51]. The assessment of RV systolic function with speckle-tracking echocardiography is less load- and angle-dependent compared to conventional two-dimensional echocardiographic parameters and more sensitive to detecting RV systolic dysfunction [52]. Two- and three-dimensional speckle-tracking echocardiography also showed a good correlation with histopathological findings of RV myocardial fibrosis and may therefore be used as a surrogate marker of RV fibrosis [53]. Prihadi et al. [52] showed that RV free wall longitudinal strain identified higher rates of RV dysfunction and improved risk stratification in patients with TR, compared to conventional echocardiographic parameters of RV systolic function (Fig. 1). Preoperative RV free wall longitudinal strain was also independently associated with outcomes in patients undergoing isolated surgery for severe functional TR [54]. Nonetheless, the evaluation of RV systolic function in patients with significant TR remains subject to afterload (which is often increased in patients with significant TR) and RV systolic function could therefore be misinterpreted. RV-pulmonary artery coupling (estimated non-invasively by the tricuspid annular plane systolic excursion/pulmonary arterial systolic pressure ratio) may overcome this limitation. After correcting for potential confounders, RV-pulmonary artery uncoupling (defined as a ratio < 0.31 mm/mmHg) was the only echocardiographic parameter that was independently associated with all-cause mortality in patients with significant TR [55]. New measures of RV systolic function, quantified with CMR, including RV shortening and effective RVEF, have been shown to better identify patients with RV dysfunction and demonstrated incremental value over conventional RVEF when evaluating outcomes [56].

Similar to patients with AS and secondary MR, the evaluation of myocardial fibrosis by late LGE-CMR has prognostic importance and could help to define the optimal timing for TR intervention [57]. However, its clinical applicability in patients with significant TR is limited by the thin-walled RV and needs to be defined in further research [53].

Recently, several transcatheter techniques have been developed, aiming to reduce TR severity. Although edge-to-edge repair is most often used, it does not directly address tricuspid annular dilation and may therefore not be applicable to all anatomies. In these situations, other devices such as annular reduction devices, spacer devices, bicaval valve implant devices, or transcatheter TV replacements could be of additional help to effectively reduce TR severity [58]. These devices have been proven to be safe and effective in reducing TR severity, and showed significant clinical improvement in patients with moderate or severe TR [59–62]. As such, these devices provide a potential future therapeutic option for inoperable and high-risk patients, although confirmation is needed from prospective, randomized controlled trials.

## Conclusion

VHD is a major health problem and is associated with increased morbidity and mortality. Treatment options are often underutilized due to increased surgical risk and late patient referral. Multimodality imaging has the potential to change the management paradigm of VHD by identifying markers of cardiac damage at an earlier stage. In addition, transcatheter valvular interventions could play a pivotal role in the treatment of patients who remain at high surgical risk or present at a later stage of their disease.

## Abbreviations

AS: Aortic stenosis
AVR: Aortic valve replacement
CMR: Cardiac magnetic resonance
EF: Ejection fraction
LGE: Late gadolinium enhancement
LV: Left ventricular
MR: Mitral regurgitation
RV: Right ventricular
TR: Tricuspid regurgitation
TV: Tricuspid valve
VHD: Valvular heart disease
